# Design and Validation of Computerized Flight-Testing Systems with Controlled Atmosphere for Studying Flight Behavior of Red Palm Weevil, *Rhynchophorus ferrugineus* (Olivier)

**DOI:** 10.3390/s21062112

**Published:** 2021-03-17

**Authors:** Maged Mohammed, Hamadttu El-Shafie, Nashi Alqahtani

**Affiliations:** 1 Date Palm Research Center of Excellence, King Faisal University, Al-Ahsa 31982, Saudi Arabia; helshafie@kfu.edu.sa (H.E.-S.); nalqahtani@kfu.edu.sa (N.A.); 2 Agricultural Engineering Department, Faculty of Agriculture, Menoufia University, Shebin El Koum 32514, Egypt; 3 Department of Crop Protection, Faculty of Agriculture, University of Khartoum, Shambat 13314, Sudan

**Keywords:** image processing, computer-assisted, graphical interface, microcontroller, flight mill, flight tunnel, flight kinematics, aerodynamic force

## Abstract

Understanding the flight characteristics of insect pests is essential for designing effective strategies and programs for their management. In this study, we designed, constructed, and validated the performance of modern flight-testing systems (flight mill and flight tunnel) for studying the flight behavior of red palm weevil (RPW) *Rhynchophorus ferrugineus* (Olivier) under a controlled atmosphere. The flight-testing mill consisted of a flight mill, a testing chamber with an automatically controlled microclimate, and a data logging and processing unit. The data logging and processing unit consisted of a USB digital oscilloscope connected with a laptop. We used MATLAB 2020A to implement a graphical user interface (GUI) for real-time sampling and data processing. The flight-testing tunnel was fitted with a horizontal video camera to photograph the insects during flight. The program of Image-Pro plus V 10.0.8 was used for image processing and numerical data analysis to determine weevil tracking. The mean flight speed of RPW was 82.12 ± 8.5 m/min, and the RPW stopped flying at the temperature of 20 °C. The RPW flight speed in the flight tunnel was slightly higher than that on the flight mill. The angular deceleration was 0.797 rad/s^2^, and the centripetal force was 0.0203 N when a RPW tethered to the end of the rotating arm. The calculated moment of inertia of the RPW mass and the flight mill’s rotating components was 9.521 × 10^−3^ N m^2^. The minimum thrust force needed to rotate the flight mill was 1.98 × 10^−3^ N. Therefore, the minimum power required to rotate the flight mill with the mean revolution per min of 58.02 rpm was approximately 2.589 × 10^−3^ W. The designed flight-testing systems and their applied software proved productive and useful tools in unveiling essential flight characteristics of test insects in the laboratory.

## 1. Introduction

The red palm weevil (RPW), *Rhynchophorus ferrugineus* (Olivier) (Coleoptera: Curculionidae), is a major internal tissue borer that causes serious economic damage to the date palm, used for both aesthetic and production values [1,2,3,4]. Globally, RPW is found in 49 countries, including 15 countries in Europe, 6 in Africa, 26 in Asia, and 2 in North America (the Caribbean). The host range of the RPW extends over 40 palm species [5]. According to some prediction models, the RPW is expected to invade more countries in the future [6]. This wide distribution and vast host range reflect the economic significance of this invasive species. The pest distribution’s temporal and geographical changes can be modeled and infested palms detected using a geographic information system (GIS) [7] depending on insect flight capacity. RPW is managed, inter alia, with chemical pesticides that negatively affect dates for export and local consumption. Additionally, the unwise use of pesticides leads to pollution of the environment and resistance to insect pests. Behavioral pest control is among the most common environmentally friendly and sustainable means of pest population suppression [1,2,4]. Knowledge about all aspects of the flight behavior of insects is necessary if we are to find ways to manage them without causing a harmful impact on the ecosystems [4,8,9]. Management technologies based on insect flight characteristics include, but are not limited to, mass trapping using pheromone-baited traps, attract and kill, and mating disruption [10,11].

Typically, two different approaches are taken to assess insect flight capacity, namely the mark-release-recapture and flight mill. The first method is to release marked insects into a field environment and then recaptured them over time at varying distances from the release point. Capture frequency and capture location of marked insects at predetermined points throughout the study are used to assess dispersal rates from a release epicenter [12]. The second method is the flight mill, where insects are tethered to an arm of a mill under controlled laboratory conditions. Of all available techniques of studying insect flight characteristics at the laboratory level, the flight mill is considered the most essential and model system [13]. Hoddle et al. used the adult of *Rhynchophorus ferrugineus* (Olivier) in computerized flight mill studies to determine this highly invasive and destructive palm pest’s flight characteristics. Such flight characteristics include velocity, flight distance covered, duration, number of flights, and cumulative flight distance. Flight mill studies have demonstrated that RPW has short-distance (<100 m) as well as long-distance fliers. RPW can fly up to 50 km in a day with predominantly diurnal flight activity [14,15]. Attisano et al. designed a simple and relatively cheap insect flight mill, which could be placed inside an incubator or environmental chamber to control flight conditions [16]. Yu incorporated sensors and software in flight mill to study the flight characteristics of the Western corn rootworm, *Diabrotica virgifera virgifera* (LeConte) [17].

Flight mill studies help understand invasive species’ flight characteristics that cannot be quantified under field conditions [18,19]. Furthermore, Lévy dispersal of insects that are involved in long-distance flight can be understood through flight mill studies [20]. Additionally, flight attributes of invasive quarantine insects could be studied through a flight mill, which is difficult to be studied by the mark-release-recapture technique [21]. Flight mill could also be used to study hormonal control of insect migration and metabolic timeline of long-distance flights [22]. Flight mill data for two other conspecific weevils; namely *R. palmarum* and *Rhynchophorus vulneratus* (Panzer), have recently been compared [23]. This information is essential to estimate the weevil’s flight capability and its dispersal potential, which are important in setting quarantine protocols, management tactics, and risk assessment [24,25]. Despite the widespread use of flight mills, they are not without shortcomings. The flight mill’s major drawback is that tethered insects do not need to generate lift as the case with free-flying insects [22]. Tethered insect must produce enough force to turn the arm of the flight mill, but this could be minimized by designing mills with little friction [26]. The tethered insect may continue to fly in the absence of tarsal contact, leading to overestimating flight potential [27]. Taylor et al. compared the flight performance of the emerald ash borer *Agrilus planipennis* on a flight mill and in free flight [28]. They found that free-flying insects have speeds three times more than tethered insects, suggesting that flight mills may underestimate the flight potential of flown insects. Thus, the flight data obtained from the flight mill may need more analysis and interpretations before being applied to field situations. Studies of insect pest biological parameters, such as feeding habits, life span, host preferences, and dispersal behavior involve several disciplines and collaborative efforts between researchers from different areas [21]. Key to the achievement of the micromechanical flying insect design is developing sensors to measure flight force [29].

Abiotic factors, particularly temperature, wind speed, and humidity, affect the flight activities of *Rhynchophorus* spp. [30,31]. Therefore, we initiated the present study with the main objective of designing, constructing, and validating computerized flight-testing mill and flight-testing tunnel systems with controlled atmosphere conditions. To help study and understand the ecology and flight behavior of the invasive red palm weevil (*Rhynchophorus ferrugineus*) as a major palm pest of global importance.

## 2. Materials and Methods

### 2.1. Architecture of Flight-Testing Systems

The computerized flight-testing mill and flight-testing tunnel systems were designed, constructed, and evaluated at the Date Palm Research Center of Excellence, King Faisal University, Saudi Arabia. The performance of the designed systems was evaluated with respect to operating accuracy and controlling ability of the testing chambers’ microclimate.

#### 2.1.1. The Computerized Flight-Testing Mill System

**Figure 1:**
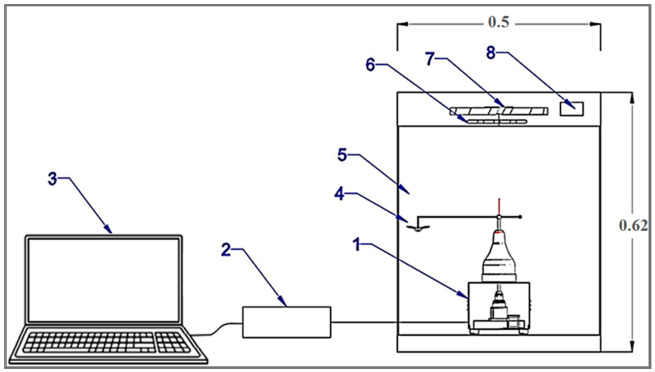
Components of the computerized flight-testing mill. (**1**) flight mill, (**2**) USB digital oscilloscope, (**3**) laptop, (**4**) tested insect, (**5**) test chamber, (**6**) heater, (**7**) fan, (**8**) temperature controller.

**Figure 2:**
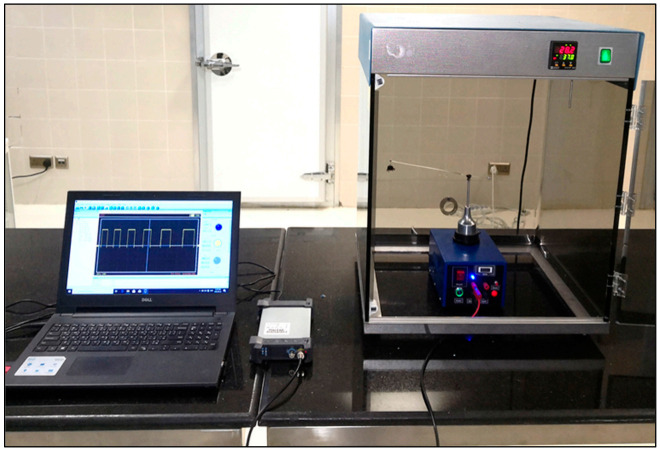
Image of the computerized flight-testing system.

This system consisted of three central units, as shown in Figure 1 and Figure 2. The first unit is the flight mill, whose function counted the number of rotations when the RPW was tethered to it and allowed to fly freely. The second unit is the testing chamber with a controlled microclimate achieved by digital temperature and relative humidity (RH) controllers. The third is the data logging and processing unit, consisting of a laptop connected with a USB digital oscilloscope (model: Hantek6022BE, Hantek Electronic Co., Ltd., Qingdao, China) with a user interface installed for real-time sampling and data processing. 

##### Design of the Flight-Testing Mill

**Figure 3:**
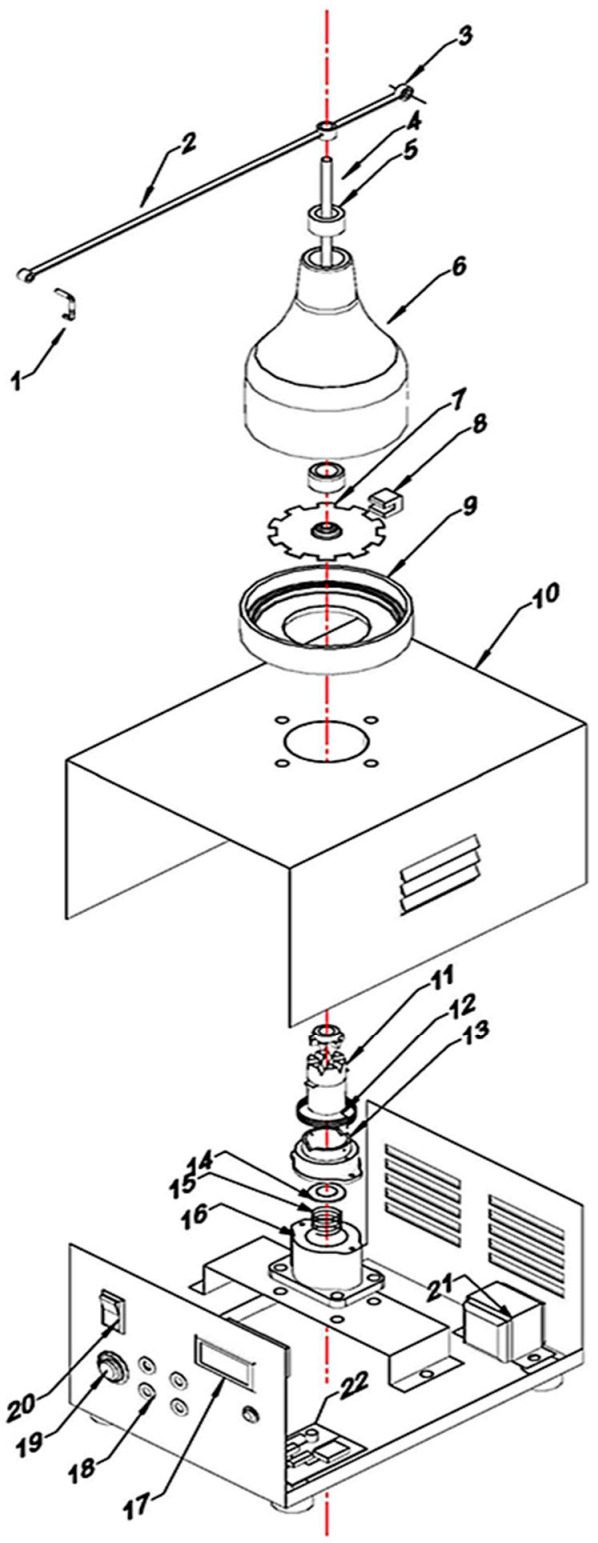
A detailed schematic diagram showing the components of the computerized flight-testing mill. (**1**) Brass connecter pole, (**2**) rotating arm, (**3**) counterweight, (**4**) spindle shaft, (**5**) brass bushing, (**6**) shaft base, (**7**) opaque plastic disc, (**8**) optoelectronic switch, (**9**) fixing base, (**10**) housing, (**11**) clutch, (**12**) spiral spring, (**13**) variable resistance, (**14**) brass flat washer, (**15**) stainless steel spiral spring, (**16**) electromagnet coil, (**17**) Liquid crystal display (LCD) of the digital counter, (**18**) signal outputs, (**19**) clutch actuation button, (**20**) power switch, (**21**) transformer, (**22**) electronic circuit of the counter.

Figure 3 shows a schematic drawing of the various components of the flight mill. The flight mill consisted of a housing box, rotating part, digital counter. The housing box dimensions having a length of 200 mm, a width of 100 mm, and a height of 100 mm with a thickness of 0.5 m, was made from galvanized iron painted by the electroplating method. The rotating part consisted of a lightweight opaque plastic disc (2 g) with a diameter of 50 mm mounted on the lightweight spindle shaft with low friction. The shaft was made from aluminum with a diameter of 4 mm. The spindle was attached with a highly flexible spring steel rotating arm with 0.5 mm thickness, 4 mm width, and a total length of 300 mm. The insect on the rotation axis of the spindle rotates the arm. The highly flexible spring steel of the rotating arm was selected to allow the insect flight force to elevate or depress the arm with vertical free-motion. The opaque disc has ten equidistant holes on its periphery.

**Figure 4:**
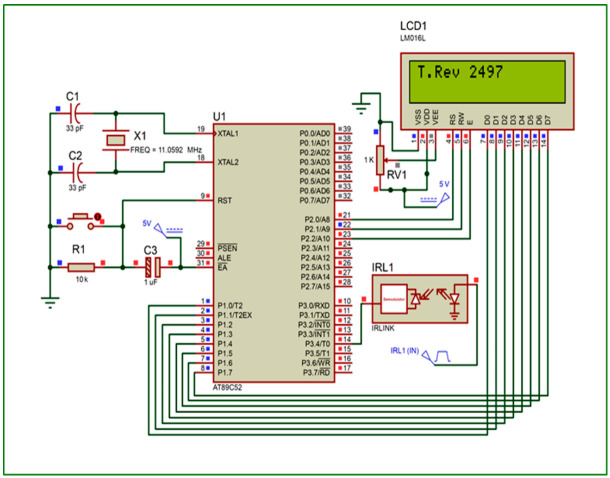
Schematic diagram of the signal counter circuit and display of the signal numbers in the Liquid crystal display (LCD).

**Figure 5:**
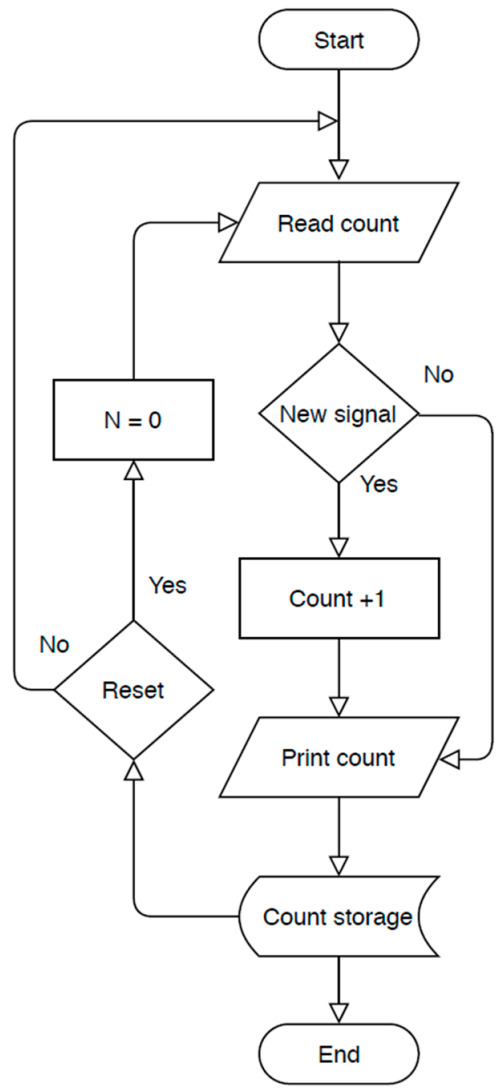
Counter flowchart to count the signal numbers.

**Figure 6:**
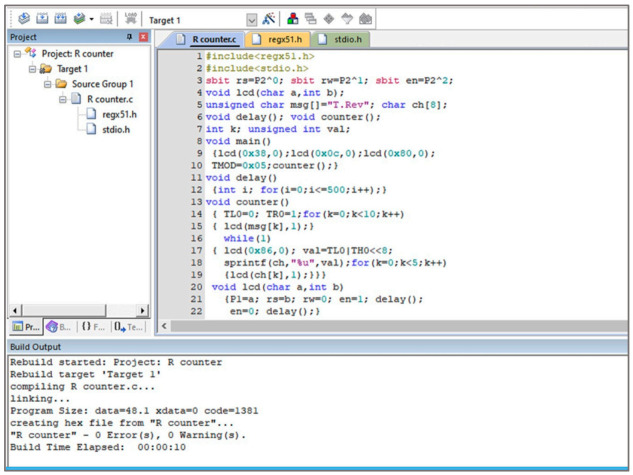
Programming code of the AT89C52 microcontroller using Keil μVision to display the signal numbers in the Liquid crystal display (LCD).

On the disc side, an optoelectronic switch (ST150, Shenzhen Sanruisheng Technology Co., Ltd., Shenzhen, China) was installed with a wide gap between the light emitter and the detector (5 mm) with insulation of ambient light and high coupling efficiency. The optoelectronic switch (ST150) was a photoelectric sensor in plastic housing containing a silicon phototransistor coupled with an infrared light-emitting diode (IR LED). The holing part of the disc can easily pass in the gap of the optoelectronic switch between the light emitter and the detector. The slot in the sensor housing provided a means of interrupting the IR with the hole of the rotating opaque disc. The phototransistor is conducted an electrical signal when a hole of the disc appears between the phototransistor and IR LED. The optoelectronic switch’s output was wired with the comparator circuit to switching the voltage output from a low to high state. The integrated circuit (IC LM339, Shenzhen Sanruisheng Technology Co., Ltd., Shenzhen, China) was used as a comparator in the circuit. Therefore, rotation of the disc resulted in a square wave at the output of IC LM339. The output of the optoelectronic switch was wired with the comparator circuit to switching the voltage output from a low to high state. The output signal of the comparator circuit was connected with the counter circuit. The liquid crystal display (LCD) of the counter was used to display numbers of the signals in the real-time of the insect flight. Figure 4 shows the signal counter’s circuit diagram, which was constructed based on the AT89C52 microcontroller. The counter algorithm was expressed as a flowchart and then the flowchart was converted into the codes of the program Figure 5. The programming of the microcontroller to displaying the count numbers in LCD was conducted using Keil μVision in C language development tools for the 8051 microcontroller family (Keil µVision5, Keil Elektronik GmbH, Munich, Germany). The circuit was simulated using the program of the Proteus design suite (Proteus 8 Professional, Labcenter Electronics Ltd., Yorkshire, England). Figure 6 shows the code used to program the AT89C52 microcontroller and display the numbers on the LCD.

##### Design of Testing Chamber

**Figure 7:**
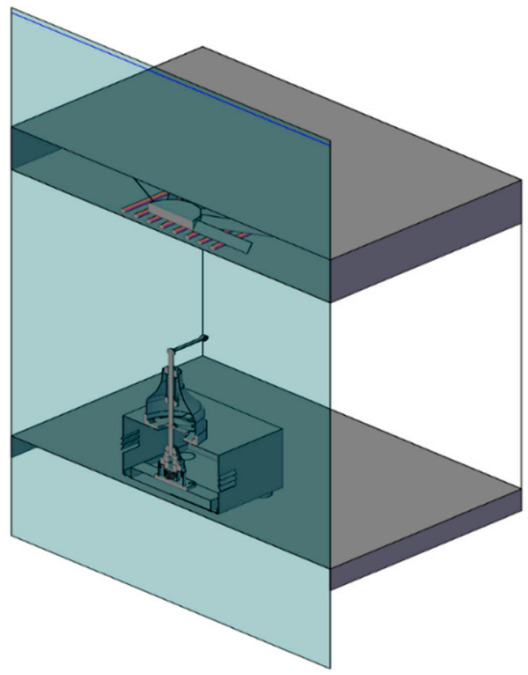
Cross-section of the testing chamber of the computerized flight mill.

Figure 7 shows a cross-section of the test chamber made of 6 mm thick polyvinyl chloride transparent sheets (PVC). The test room dimensions were 0.5 m × 0.5 m × 0.62 m for length, width, and height, respectively. The test chamber has an electronically controlled temperature using a digital temperature controller (model: STC-3018, 220 V Shenzhen ALISI Electronic Technology Co., Ltd., Shenzhen, China) and an electric air heater with a power of 300 W. To control the RH inside the testing chamber, we used an ultrasonic humidifier (model: HM3000-B5, Black & Decker, Suzhou, China) and a humidity controller (model: STC 3028, 220 V, Xuzhou Sanhe Automatic Control Equipment Co., Ltd., Xuzhou, China).

##### Data Logging and Processing

**Figure 8:**
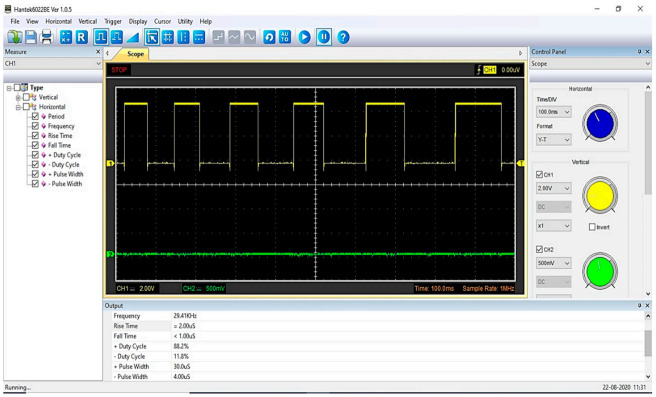
Image of waveform frequency on the user interface of the USB digital oscilloscope.

The data logging and processing unit consisted of a laptop connected with a USB digital oscilloscope (model: Hantek6022BE) with a user interface installed for real-time sampling and processing data. The comparator circuit’s output signal was connected with the USB digital oscilloscope to display waveform frequency through the user interface installed on the laptop (Figure 1). Figure 8 shows the signal’s frequency and waveform on the user interface of the USB digital oscilloscope.

**Figure 9:**
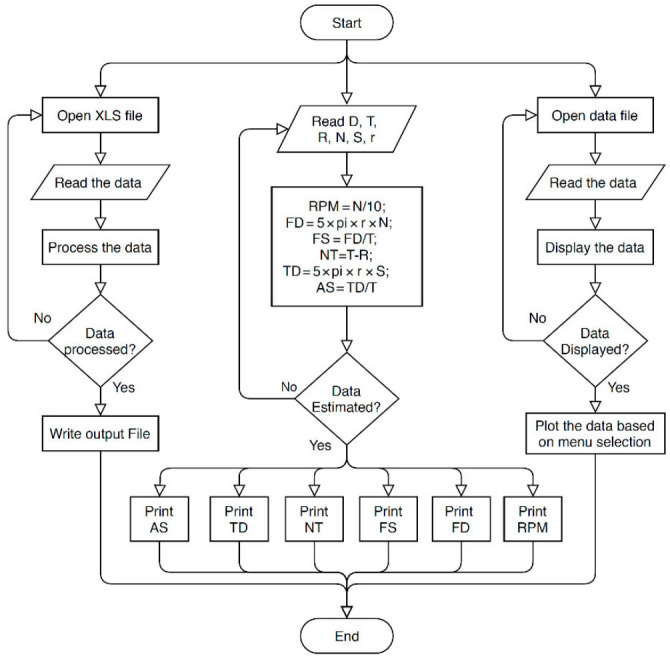
Flowchart of the implemented graphical user interface (GUI).

**Figure 10:**
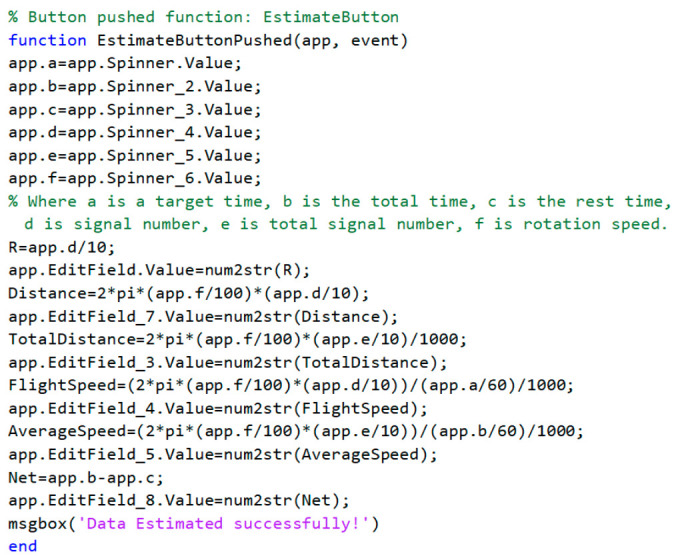
The main code of functions used for flight parameters to estimate rotation speed, flight speed, flight distance, average flight speed, total distance, and actual flight time.

For an easier understanding of the results and observation of the impact of parameters on RPW flight behavior, a graphical user interface (GUI) has been created using the App designer in the MATLAB environment (MATLAB 2020A). The GUI application was expressed as a flowchart, as shown in Figure 9. The GUI allows inputting parameters of the time, signal count, length of flight arm, and experiment information by sliders and numerical values. The main code of functions used for flight parameters estimation shown in Figure 10. After inputting the required data, the user can get the results in a required form.

Further, it is possible to import or upload an excel file containing the data. The GUI will display a graph of the flight speed, total time, or cumulative distance of the flight based on the slider’s chosen parameter. Through this GUI, the user can find directly the rotation speed, flight speed, flight distance, average of flight speed, total distance flew, and actual flight time based on the input data of target flight duration, total experimental time, rest time, rotation radius, and the number of signals recorded by the mill counter screen. The sliders and controlling buttons are grouped to improve the GUI interface and clarity of operation. The defined functionalities in the developed GUI are not complicated.

#### 2.1.2. Design of the Flight-Testing Tunnel

**Figure 11:**
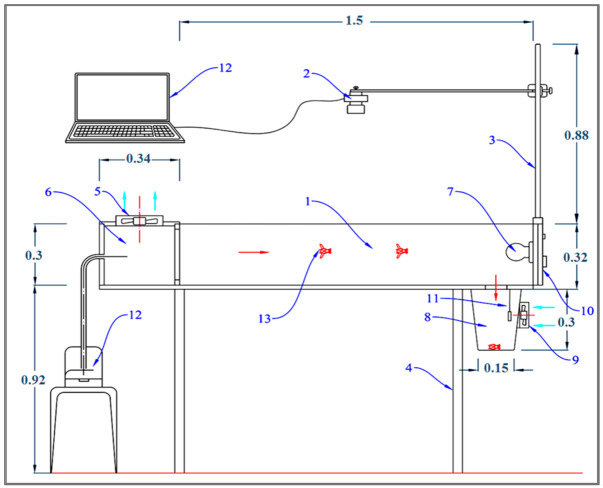
Schematic diagram of the front view of the flight-testing tunnel. (**1**) Flight tunnel made from transparent polyvinyl chloride transparent sheets (PVC), (**2**) digital camera, (**3**) holder, (**4**) metal stand, (**5**) fan for air suction, (**6**) chamber of insect, (**7**) light source, (**8**) plastic box for insect collection, (**9**) small fan for spread the pheromone scent, (**10**) control panel, (**11**) laptop (**12**) humidifier, (**13**) tested red palm red palm weevil (RPW).

**Figure 12:**
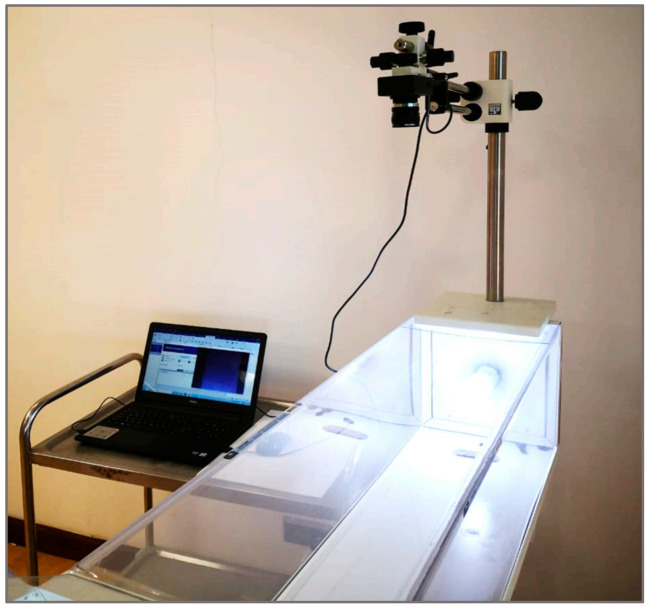
Image of the computerized flight-tunnel with the image processing system.

The flight tunnel was made from transparent PVC so that the camera can shoot clearly without hindrance, as shown in Figure 11 and Figure 12. The tunnel dimensions were 150 cm in length, 30 cm in width, and 30 cm in height. The chamber of insects was installed at a side of the tunnel, and the other side has a plastic box to collect the insects. The tunnel was supplied with light types with a light intensity of 750 Lux using compact light fluorescent bulbs with the white color of 3500 K. The idea of the flight-testing tunnel depends on the presence of a horizontal video camera to photograph the insects during flight. The velocity of flying insects was calculated from the analysis of data recorded by the camera (model: Lu075c, Lumenera CO., Ottawa, ON, Canada) with a different height ranged from 20 to 80 cm. The camera allowed remote monitoring of the activities of the treated insects through periodic analysis of videos taken by the watch camera. The captured video from the camera was transmitted to the computer for studying the insect flight speed. The free flight of the RPW was determined by taking its location differences during its flight from the chamber to pheromone scent in the collection chamber. Tracking moving RPWs was automatically followed as they move through time and space.

The programs of Image-Pro Plus 10.0.8 Image Analysis [32] and Microsoft Excel were used for numerical data analysis to determine each RPW velocity. We used this program due to its ease of use and has many features for insect tracking as an object. The basic workflow of tracking RPWs started by separating the background and objects (RPW) by thresholding each time frame into a binary image. A set limit to each RPW size and form was then conducted to reduce the false results of thresholding. The mean velocities of the RPWs over time were measured using two-dimensional (2D) tracking options. The auto-tracking tab in the program of Image-Pro contains the parameters for the tracking itself. Velocity setting determines the algorithm for searching the object in the next frame time. The test tunnel has an electronically controlled temperature using a digital temperature controller (model: STC-3018, 220 V) and an electric air heater with a power of 100 W. We used an ultrasonic humidifier (model: HM3000-B5, Black & Decker,) to control the RH inside the testing chamber and a humidity controller (model: STC 3028, 220 V).

### 2.2. Test Insects

Adults of RPW used in evaluating the efficacy of the designed systems were obtained from a colony reared in the laboratory on sugar cane and date palm bolts. The weevils to establish the colony were initially received from date palm-infested orchards (Latitude: 25.268 °N, Longitude: 49.707 °E) through insecticide-free pheromone-baited food traps. To ensure the test’s age homogeneity, cocoons were collected from the artificially infested date palm offshoot. They were kept individually in plastic cups in an incubator at a temperature of 27 °C, and 75% relative humidity under complete darkness. Weevils of both sexes (male and females) and the same age were confined together in a relatively large plastic box, provided with sugar cane, and allowed to mate. Female and male weevils were randomly selected for taking morphometric measurements before being flown on the flight-testing mill or in the flight-testing tunnel.

### 2.3. Measurements

#### 2.3.1. Temperature and Relative Humidity

**Figure 13:**
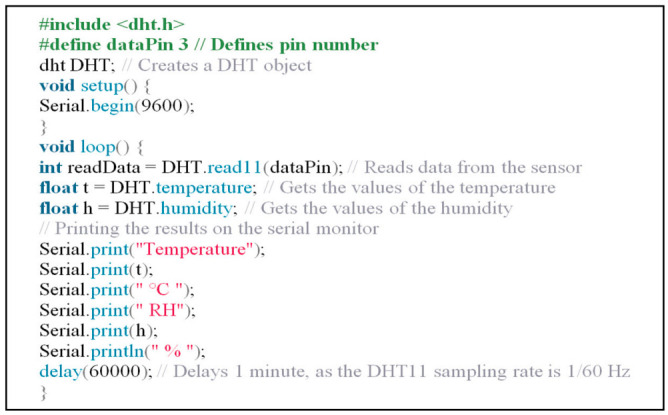
The main code used for the Arduino Uno microcontroller to monitor the temperature and relative humidity inside the flight chamber.

The DHT11 Temperature and RH sensor and Arduino Uno R3 microcontroller (Atmega 328, Shenzhen E-Best Industrial Co., Ltd., Shenzhen, China) board were used to monitor the temperature and relative humidity inside the flight chamber. The DHT11 was equipped with a calibrated digital signal output connected with the microcontroller through the digital pin 3. Figure 13 shows the code used to monitor temperature and RH inside the flight chamber and the flight tunnel.

#### 2.3.2. Flight Parameters 

The flight parameters of RPW were rotation speed, flight speed, flight distance, average flight speed, total flight distance, and actual flight time. The average velocity and acceleration of the flight mill were estimated as follows:

(1) $$v=\frac{ds}{dt}\;$$

(2) $$a\;=\;\frac{dv}{dt}dt\;$$

(3) $$\;a=\frac{\left(v_{1}\;-\;v_{0}\right)}{\left(t_{1}\;-\;t_{0}\right)}$$

where v is the average velocity of RPW (m/s), ds is flight distance (m), dt is the time taken (s), a is acceleration (m/s^2^), dv is changing in velocity (m/s), $v_{1}$ is final speed (m/s), $v_{0}$ is initial speed (m/s), $t_{1}$ is final time (s), $t_{0}$ is initial time (s).

(4) $$w=F_{g}=m\times a_{g}$$

where  w is the weight, $F_{g}$ is gravity force (N), m is the mass (kg), $a_{g}$ is the acceleration of gravity ($a_{g}=\;9.81\;m/s^{2}$).

The flight parameters were estimated directly using the created GUI based on the function shown in Figure 10.

#### 2.3.3. Force Analysis 

**Figure 14:**
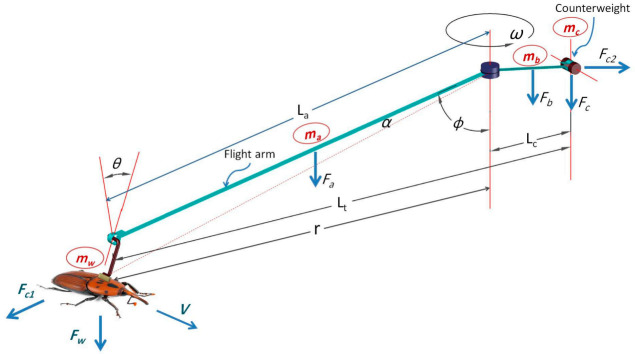
Description of flight kinematics of the red palm weevil (*R. ferrugineus*).

The rotational force was estimated based on the force demanded to maintain the rotating of the spindle shaft and its components of the flight mill at a constant speed. This was done by quickly rotating the arm of the flight mill then estimating the force based on angular deceleration of the flight arm that occurred due to the resistance torque. The spindle shaft’s rotation speed and its components and angular deceleration were estimated by analyzing the signal output by the graphs produced from the GUI. The RPW flight force measurements were calculated from the dynamics of the designed flight-mill. The flight arm was free like a seesaw to move up and down in the vertical plane. This free movement allowed the flight arm to change its angle from the resting point to the horizontal position with a maximum degree of 20° (∅° below the horizon), corresponding to the lift forces created by the flying RPW. Due to the free movement of the flight arm, the flight motion was considered a compound conical pendulum. Consequently, the aerodynamic force generated by the weevil has been estimated based on the flight arm’s angle in the vertical plane, rotation speed, and the masses of the weevil and counterweight. When the weevil mass rotates in a horizontal circle at a constant speed, the flight arm’s tension produces a vertical force and horizontal centripetal force equal to the masses’ weight, as shown in Figure 14.

The flight power of a rotating RPW was estimated based on the critical moment to rotate the flight mill using the following relations:

(5) $$P_{f}=T\times \omega$$

(6) $$T=I\times a_{a}$$

(7) $$\omega =2\;\pi \times \;N_{rps}$$

where $P_{f}$ is RPW flight power (W), T is the torque in angular motion (N m), ω is the angular velocity (rad/s), (rad = 360°/2π), *I* is the moment of inertia (kg m^2^), $a_{a}$ is angular acceleration (rad/s^2^), $N_{rps}$ is revolution per second (rev/s).

To determine the moment of inertia (*I*) around the axis passing through the center of gravity for rotating masses, the center of gravity of the system was estimated using the following relations:

(8) $$L_{x}=\frac{\sum_{i=1}^{n}m_{i}\times L_{i}}{\sum_{i=1}^{n}m_{i}}$$

(9) $$L_{x}=\frac{m_{c}\times 0+m_{b}\times 0.5\;L_{x}+m_{a}\times \left(0.5\;r+L_{x}\right)+m_{w}\times L_{t}}{m_{c}+m_{b}+m_{a}+m_{w}}$$

(10) $$r=L_{a}\sin\emptyset$$

(11) $$L_{t}=L_{a}\sin\emptyset +L_{c}$$

where $L_{x}$ is the distance between the center of gravity and counterweight (m), $m_{c}$ is the mass of counterweight (kg), $m_{b}$ is the mass of the arm length between the center of gravity and counterweight (kg), $m_{a}$ is the mass of the arm length between the center of gravity and the weevil (kg), $m_{a}$ is the mass of the weevil (kg), r is the flight radius (m), $L_{a}$ the length of the flight arm (m), ∅ is the angle of the flight arm in the vertical plane, $L_{t}$ is the distance between the RPW and the counterweight (m).

The moment of inertia is a measure of resistance to change rotation motion direction. It has the same relationship to angular acceleration as mass has to linear acceleration. It depends on the mass, shape, relative point of rotation of the object, the radius of rotation, and the distribution of mass in the body concerning the rotation axis. The moment of inertia was expressed from the total moment of inertia of all distributed masses as follow:

(12) $$I=\underset{i}{\sum }m_{i}\times L_{i}^{2}=m_{1}\times L_{1}^{2}+\;\ldots .\;+m_{n}\times L_{n}^{2}$$

where *I* is a moment of inertia (kg m^2^), m is a mass (kg), *L* is the distance between rotation mass and axis (m).

The horizontal aerodynamic force of the RPW that is required for rotating the flight-mill at a constant speed was calculated as follows:

(13) $$F_{H}=\frac{T_{r}}{L_{a}\sin\emptyset }$$

where $F_{H}$ is the horizontal aerodynamic force (N), $T_{t}$ is the total resistance torque (N m), $L_{a}$ the length of the flight arm (m), ∅ is the angle of the flight arm in the vertical plane.

Because the velocity vector of the tested RPW changes when flying in a circle, there is centripetal acceleration that was expressed as follows:

(14) $$a_{c}=\frac{v^{2}}{r}=\omega^{2}\times r$$

where $a_{c}$ is centripetal acceleration (m/s^2^), *v* is tangential velocity (m/s), *r* is flight radius (m), ω is angular velocity (rad/s). 

According to Newton’s second law, the centripetal force was expressed as follows:

(15) $$F_{C}=m\times a_{c}=m\times \frac{v^{2}}{r}=m\times \omega^{2}\times r$$

(16) $$r=L_{a}\sin\emptyset$$

where $F_{c}$ is the centripetal force (N), m is the mass (g), *r* is flight radius (m), ω is the angular velocity (rad/s), $L_{a}$ the length of the flight arm (m), ∅ is the angle of the flight arm in the vertical plane.

Based on Newton’s third law, the centripetal force ($F_{C}$) acting on the object has a centrifugal force acting in the opposite direction with the same magnitude. 

The object that moves in a circle or banked turn produces a centripetal acceleration toward the center to avoid the centrifugal thrust that tries to move it outwards. This causes stress on the RPW, because it has been forced to rotate in a circle, while it is attached perpendicular to the flight arm. The outwards thrust was reduced by inclining the connecter pole of the RPW to the outside with an angle equal to the banked angle (*θ*). The banked angle is the angle at which an RPW was inclined about its longitudinal axis concerning its rotation path. The banked angle was determined in radians and degrees using the following relations:

(17) $$\theta_{r}=\tan^{-1}\left(\frac{v^{2}}{r\times a_{g}}\right)$$

(18) $$\theta_{d}=\theta_{r}\times \left(\frac{360}{2\pi }\right)$$

where $\theta_{r}$ is the banked angle (rad), *v* is the velocity (m/s)*, r* is the radius of the circle (m)*,* $a_{g}$ is the acceleration of gravity *(*$a_{g}=\;9.81\;m/s^{2}$*)*, $\theta_{d}$ is banked angle.

#### 2.3.4. Morphometric Measurements of RPW

To determine the RPW size and other body parameters, the morphometric measurements of RPW such as thorax width, thorax thickness, and body length (from the thorax’s center-anterior margin to the center-posterior margin of the abdomen) were made using a digital caliper. The following equations were used to calculate the values of the volume and density of the weevil:

(19) $$V_{RPW}=\frac{\pi }{6}\times L_{b}\times W_{T}\times T_{T}$$

(20) $$\rho_{RPW}=\frac{M_{RPW}}{V_{RPW}}$$

where $V_{RPW}$ is the volume of RPW (mm^3^), $L_{b}$ is body length (mm), $W_{T}$ is thorax width, $T_{T}$ is thorax thickness, $\rho_{RPW}$ is the density of RPW (g/mm^3^), $M_{RPW}$ mass of RPW (g). 

The wing area was measured based on the image processing method using the open-source image-processing program (ImageJ/Fiji 1.46, LOCI, University of Wisconsin, USA). The weight of the RPW was determined using an electronic balance. 

### 2.4. Experimental Evaluation of the Flight-Testing Systems

To evaluate the flight-testing systems and studying the flight performance of RPW, four experiments were conducted at different treatments of suspension angle, temperatures, and relative humidity inside the testing chamber of the computerized flight mill system. On the other hand, an experiment was conducted to evaluate the flight performance of the RPW in the flying tunnel under different treatments of temperatures and relative humidity. All trails were carried out at the same light intensity of 750 Lux using compact light fluorescent bulbs with the white color of 3500 K because RPW flies in the daylight. The barometric pressures in the tested flight tunnel and flight chamber under different temperatures and relative humidity ranged from 0.998 to1.002 bar. 

#### 2.4.1. First Experiment: Effect of RPW Suspension Angle on Flight Speed

For conducting this experiment, RPW was glued from its thorax by the end of a small "L" shaped steel wire with a diameter of 1.5 mm and a total length of 28 mm. A small drop of glue was applied to the thorax of the insect then the tethering wire was set [23,33]. The tested weevils were held in hand until the glue dried and the wire firmly set. The other end of the wire was either connected to the flight arm vertically or tilted. The suspension angle of RPW treatments were 0, 20, 40, and 60°. The tethering method allowed the flight orientation remains in the same direction of the insect body’s long axis. The suspension angle of RPW remains fixed although the elevating and depressing of the rotating arm by insect flight force and the free vertical motion. The trial was started with a 30 min for flight test, if the flying of RPW was started during this period; the RPW is left tethered to fly for 1 h then it is released. In each treatment, three weevils were tested at the same temperature and relative humidity of 30 °C and 35%, respectively. Through this trial, the most suitable inclination angle for suspending the weevil was determined.

#### 2.4.2. Second Experiment: Effect of Temperature and Relative Humidity on Flight Parameters

This experiment aimed to determine the most relevant temperature and relative humidity for RPW flight. For conducting the trial, the RPW was suspended in the same described method in the first trial with a suspension angle of 40° that approximately equal to the calculated banked angle. The trial was started also with a 30 min for flight test, if the flying of RPW was started during this period the RPW is left tethered for 6 h. The actual flight time has been recorded in this period then the tethered RPW was released. Three weevils were tested at each treatment of temperature and relative humidity. The temperature treatments were 20 °C, 25 °C, 30 °C, 35 °C, and 40 °C (at 35% RH), whereas relative humidity treatments were 35, 50, and 65% (at 30 °C).

#### 2.4.3. Third Experiment: Flight Speed of RPW in the Flight Tunnel

To measure the insect’s flying speed in the flight tunnel, the experiments were conducted at the same treatments of temperature and relative humidity as in the second experiment. The trial aimed to compare the RPW speed at free flying versus flying speed when the RPW attached to the flight mill.

#### 2.4.4. Fourth Experiment: Force Measurements

The force measurements were conducted under the most relevant values of suspension angle (40°), temperature (30 °C), and relative humidity (35%) for RPW flying using the flight mill. Ten weevils were tested under the same treatment conditions.

### 2.5. Statistical Analysis

The experiments were arranged in randomized complete blocks and replicated three times to allow for an analysis of variance (ANOVA) of the flight parameters errors. The statistical software package IBM SPSS (SPSS Inc., Version 22, Chicago, IL, USA) was used for the analysis. Tukey multiple range tests were used to identify significantly different means within dependent variables at *p* ˂ 0.05.

## 3. Results

### 3.1. Performance Evaluation of the Systems

#### 3.1.1. Temperature and RH Control

The designed flight-testing systems were automatically controlling the testing chamber and the testing tunnel inner temperature and RH. There was no significant difference between the controlling results of temperature and RH in the flight mill’s treatment room and the flight tunnel.

**Figure 15:**
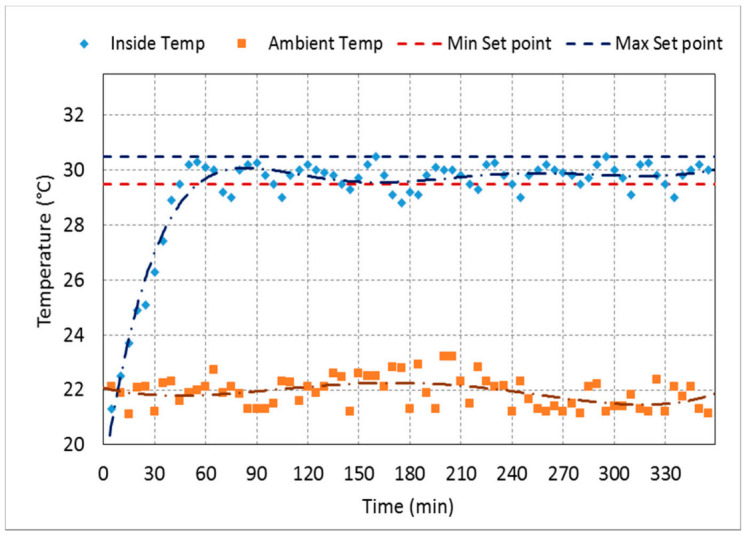
Ambient temperature, inside temperature of the testing chamber, compared to the minimum and maximum set points of 29.5 °C and 30.5 °C to obtain the target temperature of 30 °C at 35% relative humidity (RH) for 6 h.

**Figure 16:**
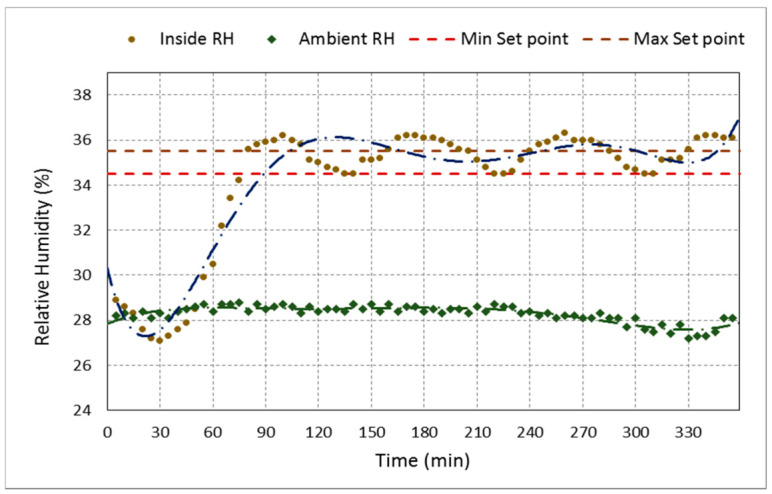
Ambient RH, inside RH of the testing chamber, compared to the minimum and maximum set points of 34.5 and 35.5% to obtain the target RH of 35% RH at 30 °C for 6 h.

Figure 15 shows the testing chamber’s actual inner temperature for a trial aimed to measure the flight parameters at a temperature of 30 °C and RH of 35% for 6 h. Figure 16 shows the actual inner RH of the testing tunnel for a trial aimed to measure the flight parameters at a temperature of 30 °C and RH of 50% for 6 h. The figures display the minimum and maximum set points and the ambient temperature and RH. The designed system automatically controls the system’s inner temperature by controlling the air heater and its fans on/off. Once the internal temperature became bigger than the minimum set point, the fan’s heat coils were turned on to bring the inner temperature back equal to the maximum set point. Then, the fan and the heating coils were turned off, and so on. The same case happened to control the inner humidity by controlling the turn on and off the ultrasonic humidifier. Consequently, as shown in Figure 15 and Figure 16 the internal temperature and RH are tightly scattered around the minimum and maximum set points.

#### 3.1.2. Graphical User Interface of the Flight Mill System

**Figure 17:**
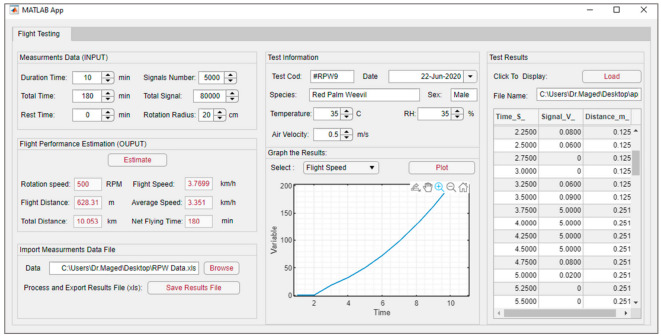
The main window of the graphical user interface (GUI) for estimation of *R. ferrugineus* flight parameters.

Figure 17 shows the main window of the implemented graphical user interface (GUI). The sliders and controlling buttons are grouped to improve the GUI interface and clarity of operation. The defined functionalities in the developed GUI performed it not complex. The GUI successfully allowed the inputting parameters of the time, signal count, length of flight arm, and experiment information by sliders and numerical values. After inputting the required data, the results in a required form were displayed. Further, it was possible to import or upload an excel file containing the data, and the GUI displayed a graph of the flight speed, total time, or cumulative distance of the flight based on the chosen parameter by the slider.

Through this GUI, the user can find directly the rotation speed, flight speed, flight distance, average of flight speed, total distance flew, and actual flight time based on the input data of target flight duration, total experimental time, rest time, rotation radius, and the number of signals recorded by the mill counter screen.

#### 3.1.3. Force Analysis of the Flight Mill

**Figure 18:**
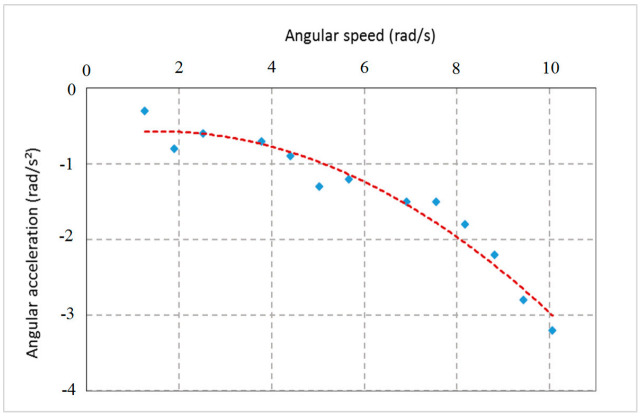
Relationship between the angular speed and angular acceleration of the designed flight mill.

Figure 18 presents the experimental measurement of the relationship between the rotating arm’s angular speed and the angular deceleration of the computerized flight mill. The data presented are for the designed flight mill tested in a controlled atmosphere with a temperature of 30 °C and RH of 35% and the suspension angle of RPW was 40°. The deceleration occurred due to the resistance of the rotation shaft to rotate. The relationship was used to determine the flight force that the RPW must overcome to maintain the flight mill rotating at a constant speed. For the mean flight speed measured of the tested weevils of 81.98 m/min (6.08 rad/s), the angular deceleration was 0.797 rad/s^2^ when a RPW tethered to the end of the rotating arm. The centripetal force at the mean flight speed for the flight arm with a tethered RPW was 0.0203 N. The calculated moment of inertia of the RPW mass and the rotating components of the flight mill was 9.521 × 10^−3^ N m^2^. The minimum thrust force needed to rotate the flight mill was 1.98 × 10^−3^ N. Therefore, the minimum power required to rotate the flight mill with the mean revolution per min of 58.02 rpm was approximately 2.589 × 10^−3^ W.

#### 3.1.4. Tracking of RPW in the Flight Tunnel

**Figure 19:**
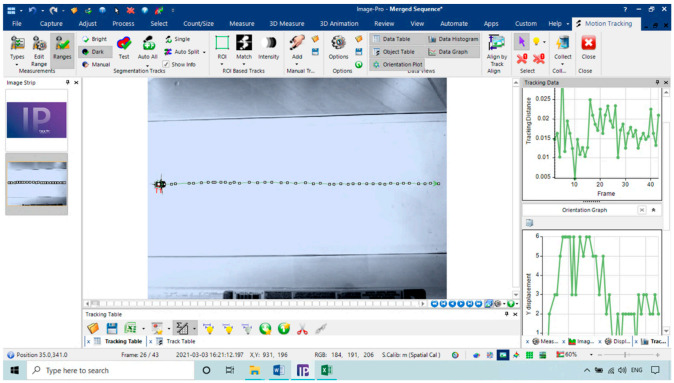
The main window of the Image-Pro program (Image-Pro Plus, Version 10.0.8 for Windows 10).

**Figure 20:**
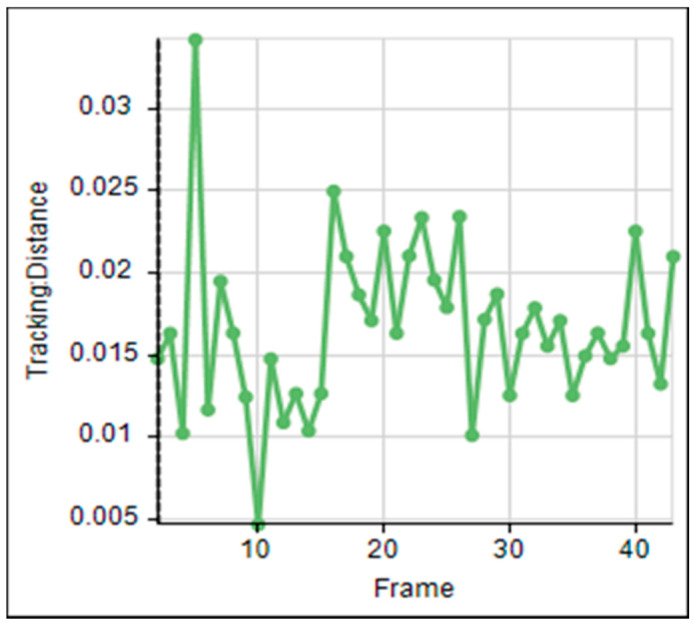
The relation between the frame and tracking distance of the insect (m).

**Figure 21:**
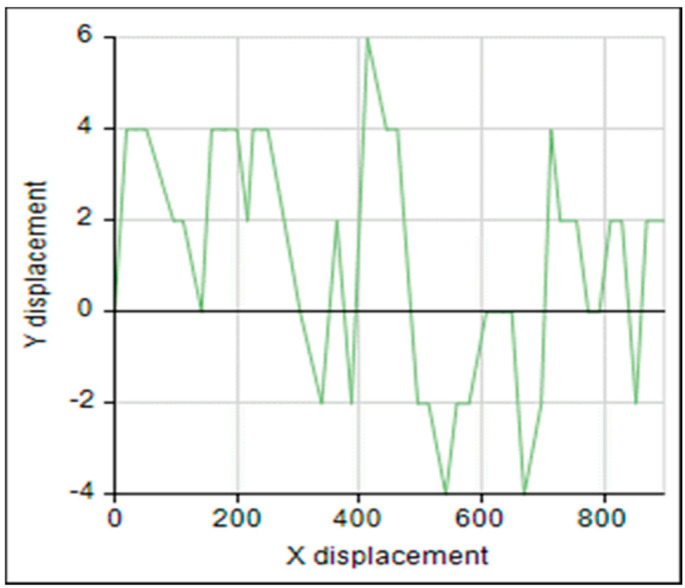
X displacement versus Y displacement of *R. ferrugineus* tracking direction.

The flight tunnel was designed to investigate the effects of different temperature and relative humidity parameters on the insect flight with free motion and compare the flight speed with the flight mill under the same parameters. The program Image-Pro (Image-Pro Plus, Version 10.0.8 for Windows) was used to follow the insect. The tracking feature was used to track the insect as an object when it flies through time and space. The tracking data were saved and sent to the Excel spreadsheet for statistical analysis and show the flight speed graphs. Figure 19 shows the main window of the Image-Pro program. The tracking data of the image analysis was saved and sent to the Excel spreadsheet. The objects were identified from the background to track them by setting the darker pixels as objects, and brighter pixels as background. The frame rate of the used camera was set to 60 f/s. The camera covered a distance of 0.95 m of the flight tunnel, and the insect took 0.71 ± 0.2 s to cross this distance. Therefore, the average speed was 1.39 ± 0.28 m/s. This program successfully analyzed the images and gave direct results for tracking the insect, as shown in Figure 19. Figure 20 shows the relation between the frame and tracking distance (m). The average motion distance in the frame was 0.023 ± 0.01 m, and the frame time was 0.0167 s. Figure 21 shows the X displacement versus Y displacement of the RPW as an indicator of the direction of movement. From this figure, it can be observed that the flight of the RPW was in a straight direction in the flight-testing tunnel.

### 3.2. Flight Behavior of the RPW

#### 3.2.1. Morphometric Measurements of RPW

Table 1 presents some morphometric measurements of weevils male and female used in the study. There was a significant difference between the mass of RPW male and female (RM ANOVA, F_1, 19_ = 11.51, *p* = 0.003), while there are no statistically significant differences for the other morphometric measurements.

#### 3.2.2. Effect of RPW Suspension Angle on Flight Speed

**Figure 22:**
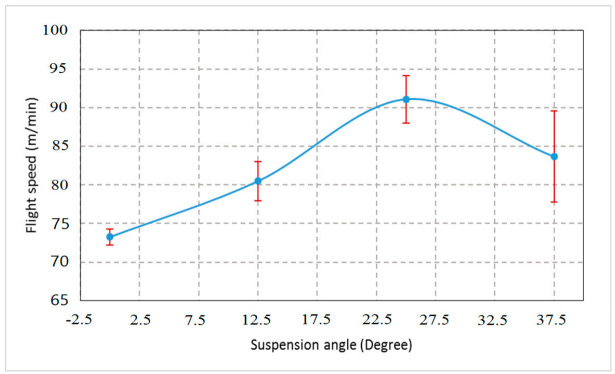
Effect of RPW suspension angle on flight speed of RPW under a controlled atmosphere with temperatures of 30 °C and RH of 35%.

**Figure 23:**
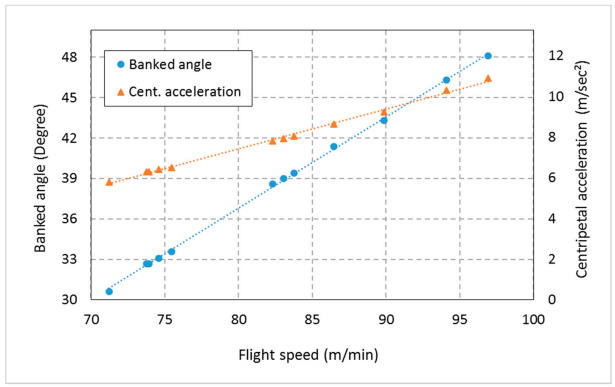
Flight speed versus calculated banked angle and centripetal acceleration of ten weevils, measured at suspension angle of 40° under a controlled atmosphere with temperatures of 30 °C and RH of 35%.

The weevils have achieved normal flight throughout the range of tested suspension angles of 0, 20, 40, and 60° under temperatures of 30 °C and RH of 35% as shown in Figure 22. However, there was a significant difference between the average flight speed of the RPW at the tested angels (RM ANOVA, F_3, 11_ = 4.24, *p* = 0.046). There were no significant differences between the means of flight speeds of the tested males and females weevils. The mean flight speed of RPW was 82.12 ± 8.5 m/min. The highest flight speed of the RPW was recorded at a suspension angle of 40° degrees (91.09 ± 5.33 m/min), which approximates the calculated banked angle. The banked angle depends on the flight speed and the length of the flight arm. Figure 23 shows the RPW flight speed versus calculated banked angle and centripetal acceleration for ten weevils at a suspension angle of 40°. The mean banked angle was 38.23 ± 1.67°, while the minimum and maximum degrees were 30.6 and 48.1°, respectively. The mean centripetal acceleration was 7.85 ± 0.48°, while the minimum and maximum degrees were 5.8 and 10.9, respectively. Based on the results obtained in this experiment, the RPW was suspended in all experiments at an angle of approximately 40°. As there were no significant differences between males and females in flight speed, only males were used in all experiments.

#### 3.2.3. Effect of Temperature and Relative Humidity on Flight Parameters

Table 2 shows the effect of temperature on flight parameters of RPW at RH of 35% using the computerized flight mill. There was a significant difference between the average revolution per sec of the flight arm (RM ANOVA, F_4, 14_ = 97.39, *p* < 0.01), flight speed (RM ANOVA, F_4, 14_ = 97.39, *p* < 0.01), cumulative flight time (RM ANOVA, F_4, 14_ = 90.5, *p* < 0.01), and the cumulative flight distance (RM ANOVA, F_4, 14_ = 226.8, *p* < 0.01) of the weevils under different temperature treatments. RPW stopped flying at the temperature of 20 °C, while a slight flight of some weevils was noted at 40 °C. Based on the results, the temperature of 30 °C is considered the most suitable temperature for flying the weevils.

Table 3 shows the effect of RH on the flight parameters of RPW at a temperature of 30 °C using the computerized flight mill. The revolution per sec of the flight arm (RM ANOVA, F_2, 8_ = 0.31, *p* = 0.75), flight speed (RM ANOVA, F_2, 8_ = 0.31, *p* = 0.75), cumulative flight time (RM ANOVA, F_2, 8_ = 0.49, *p* = 0.63), and the cumulative flight distance (RM ANOVA, F_2, 8_ = 0.65, *p* = 0.55) of the weevils did not differ significantly under different treatments of RH. Based on the obtained results, 35% RH was applied in the experiments.

#### 3.2.4. Flight Speed in the Flight Tunnel

**Figure 24:**
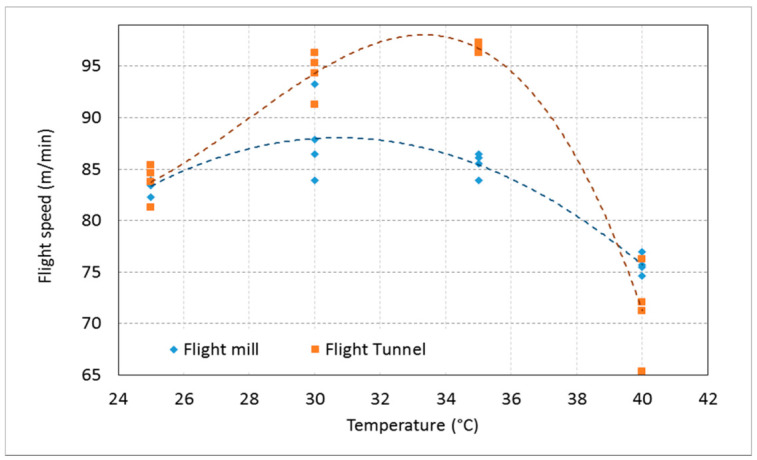
Effect of temperature on flight speed of RPW at RH of 35% using the computerized flight mill and the flight tunnel.

RPW exhibited normal flight speed throughout the tested temperatures from 25 °C to 40 °C at 35% RH in the flight tunnel. There was a significant difference between the flight speed (RM ANOVA, F_1,__9_ = 1273, *p* < 0.001) of the weevils under applied temperature treatments, while there was no significant difference between the temperature of 30 and 35 °C. RPW stopped flying at the temperature of 20 °C, and the flight speed of some weevils at a temperature of 40 °C was decreased. The results showed that the flying speed of the weevil in the flight tunnel was slightly higher than the flight speed of the flight mill, so this must be taken into account when using the fly mill for other insects. Due to the significant difference in RPW flight speed between the flight mill and the flight tunnel (RM ANOVA, F_1,__9_ = 7.861, *p* = 0.011). Figure 24 shows the effect of temperature on flight speed of RPW at RH of 35% using the computerized flight mill and the flight tunnel. There was no significant difference in the effect of RH on the flight speed of the tested weevils between the flying tunnel and the flying mill. The relationship between temperature treatments and flight speed of the tested weevils in the flight mill is expressed by the following regression equation (R^2^ = 0.8589).

y = − 0.1433x^2^ + 8.8025x − 47.164 (21)

The relationship between temperature treatments and flight speed of the tested RPWs in the flight tunnel is expressed by the following regression equation (R^2^ = 0.95)

y = − 0.0263x^3^ + 2.2082x^2^ − 59.414x + 600.6 (22)

## 4. Discussion

Knowledge of invasive species’ flight capabilities is essential for delineating quarantine boundaries and developing sound strategies and tactics for their management. However, gaining such information for these species in the field and natural habitat is very difficult and resource-demanding. Suitable technologies and tools to study insects’ long-distance flights in the fields are not available [34]. Insect flight propensity (distance flown, speed, duration) obtained by flight mill studies could give good information about the flight performance of insects. In this respect, this study presented a detailed description of the computer-monitored flight-testing systems’ design, construction, and performance evaluation measurements. The systems description covered all mechanical, electronic, software, and instrumentation details to facilitate these systems’ reproduction in a final product form.

The systems provide fully automated data processing of the most dangerous insect’s flight performance on the date palm. Moreover, the systems were designed to fit with different sizes of the tested RPW in their hardware and software aspects under a controlled atmosphere in both flight mill chamber or flight tunnel.

The present investigation revealed that flight parameters of RPW, such as flight velocity, cumulative flight time, and cumulative flight distance have shown significant differences under different temperature treatments. The test weevils stopped flight at a temperature of 20 °C. This has implications for managing this RPW and palm services, particularly frond pruning that necessitates creating a wound in the palm trunk that could be carried out in the wintertime when ambient temperatures are well below this fight activity threshold. Temperature and humidity are two important environmental variables that greatly affect weight loss and flight propensity in *Rhynchophorus cruentatus* (F.). Flight activity increases significantly as temperature increases and humidity declines [30].

In our 6-h trials, the RPW can fly a maximum distance of 23.59 km on the flight mill at a temperature of 30 °C and 35% RH. Hoddle et al. found that the weevil can fly a maximum distance of 60–70 km in 24-h [15], while Avalos et al. found that the maximum distance flown by the RPW in 12-h flight trials was 19.6 km [35]. The cumulative lifetime distance for the male of RPW permitted to fly for 3h per trial was approximately 315 km in multiple repeated flights [25]. This reflects the great potential of the red palm RPW to fly over long distances and its ability to dispersal in palm groves. However, Maes et al. stated that a flight mill might lead to overestimation of an insect’s dispersal capacity compared to a free-flying insect because of the absence of air resistance and lack of tarsal contact [36].

In contrast to this idea, some authors think that the flight mill underestimates the flight potential of tethered insects because of the initial moment of inertia when the insect accelerates the rotating arm of the flight mill [25,33]. In this study, we reported a calculated moment of inertia of the weevil mass and the flight mill’s rotating components of 9.521 × 10^−3^ N m^2^. Accordingly, the minimum thrust force needed by the weevil to rotate the flight mill is 1.98 × 10^−3^ N. For RPW to fly at a constant flight speed, it needs to create more force to counter the resistance torque of the flight-mill (due to friction of the rotating shaft and air resistance). The RPW needs energy in terms of ATP to move the arm of the flight mill. The required energy is obtained by oxidation of fat, which leads to a reduction of weight and finally, death of the flown weevils. In this respect, Hoddle et al. in a 24-h trial period recorded around 80% of weevils death that has flown over a distance of 25–35 km [15]. This indicates the long flight cost in terms of body weight loss and reduction in survival rates of the invasive species.

The experimental setup, the weevil’s physiological status, and the design of the flight mill (hardware and software) make it difficult to compare the results obtained by the different studies [33]. Our results have shown that the weevil’s highest flight speed (91.09 ± 5.33 m/min) was recorded at a suspension angle of 40° at a temperature of 30 °C and 35% RH, which approximates the calculated banked angle. Barkan et al. conducted experiments on *R. ferrugineus* to estimate the weevil dispersal potential through repeated flights on flight mill. They stated that tethering the RPW at 40° of body roll was close to free flight but require high-energy demand from the tethered insect [25]. In this respect, Hoddle et al. reported the highest velocity of *R. ferrugineus* in the summer of 107.40 m/min. They added that the highest speed of the RPW was attained in summer, followed by spring and winter. The season of the year was found to influence the total distance flown by RPW more profoundly than the gender of the RPW [15].

Our results revealed that there were no differences in flight parameters (total flight distance and flight velocity) of mated weevils of both sexes. These results are in line with the finding of other authors who studied the flight behavior of the red palm RPW using a flight mill [15,25,35,37]. The absence of sex-biased flight potential might be necessary for the establishment of new colonies of an invasive species [25]. The in-grove dispersal and spread of the red palm RPW depend largely on short-distance flight activities, which are, in turn, influenced by environmental factors, such as temperature and humidity. The establishment of the RPW colonies, after the initial introduction, requires the movement of gravid females or the being together of the females and males. 

The relationship between the RPW free flight characteristics and flight attributes on the flight mill is yet to be evaluated [33]. In the present investigation, we attempted to study the flight characteristics of RPW on the flight-testing mill and in a flight-testing tunnel with conditions simulating the field environment including light intensity, temperature, humidity, and wind current. The comparison we made between the flight performance of RPW on the flight mill and in the flight tunnel indicated that the flight velocity of the RPW was faster in the tunnel where the RPW had the chance to fly freely. No studies are available in the literature concerning free flight of red palm RPW; however, trials on the desert locust, *Schistocerca gregaria* revealed that free-flying swarming locusts have higher wing beat frequencies than those in the laboratory [38]. On the other hand, Taylor et al. found that the free-flying emerald ash borer’s speed, *Agrilus planipennis* was three times greater than the tethered beetle [28]. More future studies on flight-testing tunnels or chambers are needed to unveil flight characteristics of the red palm RPW in an environment simulating actual field conditions.

## 5. Conclusions

Understanding the flight behavior of major coleopteran pests of the date palm, particularly the invasive RPW, is essential for designing effective management strategies against them. We designed, constructed, and evaluated modern flight-testing systems’ effectiveness for studying RPW behavior under controlled atmosphere conditions. The designed systems and their applied software proved to be mechanically sound, productive, and simple to operate with high accuracy. Thus, the computerized flight systems facilitate studying insect behavior in the laboratory under a controlled atmosphere. Such studies reduce the effort exerted and the cost of deploying traps in the field and help overcome the seasonality and the rare appearance of adult insects. Besides, monitoring insect behavior in the lab is more effortless and fast. Further studies are certainly needed for testing and evaluating the flight system at a larger scale for various pests and conditions.

## Figures and Tables

**Table 1:** Mean and standard deviation (SD) of some morphometric measurements of weevils used in the study.

Characteristic	RPW Sex			
	Female		Male	
	Mean	SD	Mean	SD
Mass (g)	1.13 ^A^	0.17	0.92 ^B^	0.09
Body length (mm)	25.76 ^A^	2.69	26.26 ^A^	1.01
Thorax width (mm)	10.96 ^A^	0.76	10.86 ^A^	0.57
Thorax thickness (mm)	8.56 ^A^	0.50	8.50 ^A^	0.82
Calculated volume (cm^3^)	1.27 ^A^	0.23	1.28 ^A^	0.21
Density (g/cm^3^)	0.91 ^A^	0.21	0.73 ^A^	0.11
Wing length (mm)	22.03 ^A^	1.33	21.88 ^A^	1.13
Wings area (mm^2^)	219.1 ^A^	15.31	218.9 ^A^	13.27

Figures with the same letter horizontally for each parameter are non-significant at alpha values of 0.05. The data presented above indicate the mean of 10 *R. ferrugineus*.

**Table 2:** Effect of temperature on flight parameter of RPW at RH of 35% using the computerized flight mill.

Flight Parameter	Temperature (°C)				
	20	25	30	35	40
Revolution per sec (RPS)	0	0.94 ±0.08 ^A^	1.04 ± 0.56 ^A^	0.93 ± 0.13 ^A^	0.89 ± 0.02 ^A^
Flight speed (m/min)	0	79.69 ± 7.3 ^A^	87.89 ± 4.8 ^A^	78.85 ± 11.1 ^A^	76.02 ± 1.7 ^A^
Cumulative flight time (min)	0	273.6 ± 6.4 ^A^	268.7 ± 10.1 ^A^	219.7 ± 45.4 ^A^	98.3 ± 12.0 ^B^
Cumulative flight distance (km)	0	21.82 ± 2.2 ^A^	23.59 ± 0.5 ^A^	16.98 ± 0.87 ^B^	7.44 ± 0.76 ^C^

Figures with the same letter horizontally for each parameter are non-significant at alpha values of 0.05.

**Table 3:** Effect of relative humidity on flight parameter of RPW at a temperature of 30 °C using the computerized flight mill.

Flight Parameter	Relative Humidity (%)		
	35	55	75
Revolution per sec (RPS)	0.95 ±0.05 ^A^	0.97 ± 0.07 ^A^	0.99 ± 0.06 ^A^
Flight speed (m/min)	80.54 ± 4.4 ^A^	81.95 ± 5.7 ^A^	83.93 ± 5.56 ^A^
Cumulative flight time (min)	222.0 ± 31.1 ^A^	240.0 ± 15.3 ^A^	226.7 ± 15.3 ^A^
Cumulative flight distance (km)	17.89 ± 2.89 ^A^	19.61 ± 1.14 ^A^	18.98 ± 0.91 ^A^

Figures with the same letter horizontally for each parameter are non-significant at alpha values of 0.05.

## Data Availability

Not applicable.

## References

[1] El-Shafie H.A.F., Mohammed M.E.A. (2016). Description and quantification of damage incurred by the longhorn date palm stem borer Jebusaea hammerschmidti Reiche, 1877 (Coleoptera: Cerambycidae) on date palm (Phoenix dactylifera Linnaeus, 1753). Int. J. Entomol. Res..

[2] Milosavljević I., El-Shafie H.A.F., Faleiro J.R., Hoddle C.D., Lewis M., Hoddle M.S. (2019). Palmageddon: The wasting of ornamental palms by invasive palm weevils, *Rhynchophorus* spp.. J. Pest Sci..

[3] El-Shafie H.A.F. (2019). The use of phosphine as curative treatment against date palm borers. Outlooks Pest Manage..

[4] El-Shafie H.A., Mohammed M.E., Sallam A.K.A. (2020). Quarantine protocol against coleopteran borers in date palm offshoots using eco2fume gas. Outlooks Pest Manage..

[5] EPPO Rhynchophorus ferrugineus. EPPO datasheets on pests recommended for regulation. https://gd.eppo.int.

[6] Fiaboe K.K.M., Peterson A.T., Kairo M.T.K., Roda A.L. (2012). Predicting the potential worldwide distribution of the red palm weevil Rhynchophorus ferrugineus (Olivier) (Coleoptera: Curculionidae) using ecological niche modeling. Florida Entomol..

[7] Massoud M., Faleiro J., El-Saad M., Sultan E. (2011). Geographic information system used for assessing the activity of the red palm weevil Rhynchophorus ferrugineus (Olivier) in the date palm Oasis of Al-Hassa, Saudi Arabia. J. Plant Prot. Res..

[8] Maged E.A.M., Hamadttu A.F.E.-S., Mohammed R.A. (2020). Recent Trends in the Early Detection of the Invasive Red Palm Weevil, Rhynchophorus ferrugineus (Olivier). Invasive Species—Introduction Pathways, Economic Impact, and Possible Management Options.

[9] El-Shafie H.A.F., Abdel-Banat B.M.A., Mohammed M.E.A., Al-Hajhoj M.R. (2019). Monitoring tools and sampling methods for major date palm pests. CAB Rev. Perspect. Agric. Vet. Sci. Nutr. Nat. Resour..

[10] Vanhove W., Feng Y., Yu M., Hafiz I.O., Vanhoudt N., Van Damme P.L.J., Zhang A. (2020). Evaluation of attract-and-kill strategy for management of cocoa pod borer, Conopomorpha cramerella, in Malaysia cocoa plantation. Int. J. Pest Manage..

[11] Wijayaratne L.K.W., Burks C.S. (2020). Persistence ofmating suppression of the indianmeal moth plodia interpunctella in the presence and absence of commercialmating disruption dispensers. Insects.

[12] Margaritopoulos J.T., Voudouris C.C., Olivares J., Sauphanor B., Mamuris Z., Tsitsipis J.A., Franck P. (2012). Dispersal ability in codling moth: Mark-release-recapture experiments and kinship analysis. Agric. For. Entomol..

[13] Schumacher P., Weyeneth A., Weber D.C., Dorn S. (1997). Long flights in Cydia pomonella L. (Lepidoptera: Tortricidae) measured by a flight mill: Influence of sex, mated status and age. Physiol. Entomol..

[14] Faleiro J.R., Ashok Kumar J., Rangnekar P.A. (2002). Spatial distribution of red palm weevil Rhynchophorus ferrugineus Oliv. (Coleoptera: Curculionidae) in coconut plantations. Crop Prot..

[15] Hoddle M.S., Hoddle C.D., Faleiro J.R., El-Shafie H.A.F., Jeske D.R., Sallam A.A. (2015). How Far Can the Red Palm Weevil (Coleoptera: Curculionidae) Fly?: Computerized Flight Mill Studies with Field-Captured Weevils. J. Econ. Entomol..

[16] Attisano A., Murphy J.T., Vickers A., Moore P.J. (2015). A simple flight mill for the study of tethered flight in insects. J. Vis. Exp..

[17] Yu E.Y., Gassmann A.J., Sappington T.W. (2019). Using flight mills to measure flight propensity and performance of western corn rootworm, diabrotica virgifera virgifera (Leconte). J. Vis. Exp..

[18] Lopez V.M., McClanahan M.N., Graham L., Hoddle M.S. (2014). Assessing the Flight Capabilities of the Goldspotted Oak Borer (Coleoptera: Buprestidae) with Computerized Flight Mills. J. Econ. Entomol..

[19] Naranjo S.E. (2019). Assessing insect flight behavior in the laboratory: A primer on flight mill methodology and what can be learned. Ann. Entomol. Soc. Am..

[20] Wiman N.G., Walton V.M., Shearer P.W., Rondon S.I., Lee J.C. (2015). Factors affecting flight capacity of brown marmorated stink bug, Halyomorpha halys (Hemiptera: Pentatomidae). J. Pest Sci..

[21] Martí-Campoy A., Ávalos J.A., Soto A., Rodríguez-Ballester F., Martínez-Blay V., Malumbres M.P. (2016). Design of a computerised flight mill device to measure the flight potential of different insects. Sensors.

[22] Minter M., Pearson A., Lim K.S., Wilson K., Chapman J.W., Jones C.M. (2018). The tethered flight technique as a tool for studying life-history strategies associated with migration in insects. Ecol. Entomol..

[23] Hoddle M.S., Hoddle C.D., Milosavljević I. (2020). How Far Can Rhynchophorus palmarum (Coleoptera: Curculionidae) Fly?. J. Econ. Entomol..

[24] Soroker V., Suma P., La Pergola A., Llopis V.N., Vacas S., Cohen Y., Cohen Y., Alchanatis V., Milonas P., Golomb O., Soroker V., Colazza S. (2017). Surveillance Techniques and DetectionMethods for Rhynchophorus ferrugineus and Paysandisia archon. Handbook of Major Palm Pests: Biology and Management.

[25] Barkan S., Hoffman A., Hezroni A., Soroker V. (2018). Flight Performance and Dispersal Potential of Red Palm Weevil Estimated by Repeated Flights on Flight Mill. J. Insect Behav..

[26] Jones H.B.C., Lim K.S., Bell J.R., Hill J.K., Chapman J.W. (2016). Quantifying interspecific variation in dispersal ability of noctuid moths using an advanced tethered flight technique. Ecol. Evol..

[27] Edwards J.S. (2006). The central nervous control of insect flight. J. Exp. Biol..

[28] Taylor R.A.J., Bauer L.S., Poland T.M., Windell K.N. (2010). Flight performance of agrilus planipennis (Coleoptera: Buprestidae) on a flight mill and in free flight. J. Insect Behav..

[29] Wood R.J., Fearing R.S. (2001). Flight force measurements for a micromechanical flying insect. IEEE Int. Conf. Intell. Robot. Syst..

[30] Weissling T.J., Giblin-Davis R.M., Center B.J., Hiyakawa T. (1994). Flight behavior and seasonal trapping of Rhynchophorus cruentatus (Coleoptera: Curculionidae). Ann. Entomol. Soc. Am..

[31] Ávalos J.A., Balasch S., Soto A. (2016). Flight behaviour and dispersal of Rhynchophorus ferrugineus (Coleoptera: Dryophthoridae) adults using mark-release-recapture method. Bull. Entomol. Res..

[32] MediaCybernetics Image-Pro Plus. https://www.mediacy.com/imagepro.

[33] Ribak G., Barkan S., Soroker V. (2017). The aerodynamics of flight in an insect flight-mill. PLoS ONE.

[34] Daniel Kissling W., Pattemore D.E., Hagen M. (2014). Challenges and prospects in the telemetry of insects. Biol. Rev..

[35] Ávalos J.A., Martí-Campoy A., Soto A. (2014). Study of the flying ability of Rhynchophorus ferrugineus (Coleoptera: Dryophthoridae) adults using a computer-monitored flight mill. Bull. Entomol. Res..

[36] Maes S., Massart X., Grégoire J.C., De Clercq P. (2014). Dispersal potential of native and exotic predatory ladybirds as measured by a computer-monitored flight mill. BioControl.

[37] Hoddle M.S., Hoddle C.D., Milosavljević I. (2021). Quantification of the Life Time Flight Capabilities of the South American Palm Weevil, Rhynchophorus palmarum (L.)(Coleoptera: Curculionidae). Insects.

[38] Baker P.S., Cooter R.J. (1979). The natural flight of the migratory locust, Locusta migratoria L.—I. Wing movements. J. Comp. Physiol..

